# Targeting Cytokines in GVHD Therapy

**Published:** 2017-06-28

**Authors:** Sandeep Kumar, Hemn Mohammadpour, Xuefang Cao

**Affiliations:** 1 Department of Immunology, Roswell Park Cancer Institute, Buffalo, NY 14263, USA

**Keywords:** Hematopoietic cell transplantation, Graft-versus-host disease, T cells, Cytokines

## Abstract

Transplantation of donor-derived allogeneic hematopoietic cells causes increased survival in patients suffering from various blood cancers and other hematologic and immunologic diseases. However, this health benefit is limited to certain patients. One major complication is graft-versus-host disease (GVHD) that occurs when donor-derived immune cells recognize host cells/tissues as foreign and perpetrate subsequent destruction. Cytokines are a major class of effector molecules that are involved in GVHD pathogenesis. Proinflammatory cytokines released by activated immune cells including T cells lead to the onset of GVHD. T cell depletion (TCD) is an effective approach for GVHD prevention. Several immune suppressive drugs are also used to treat GVHD. However, these prophylactic and treatment strategies often lead to an immune compromised state that increases the risk for infection and cancer relapse. Considering the adverse effects of TCD and overall immune suppression, more selective managements such as approaches targeting proinflammatory cytokines have emerged as a promising strategy to control GVHD. Therefore, this work is dedicated to review recent development in the studies of cytokines and their future implication in GVHD therapy.

## INTRODUCTION

**Figure 1:**
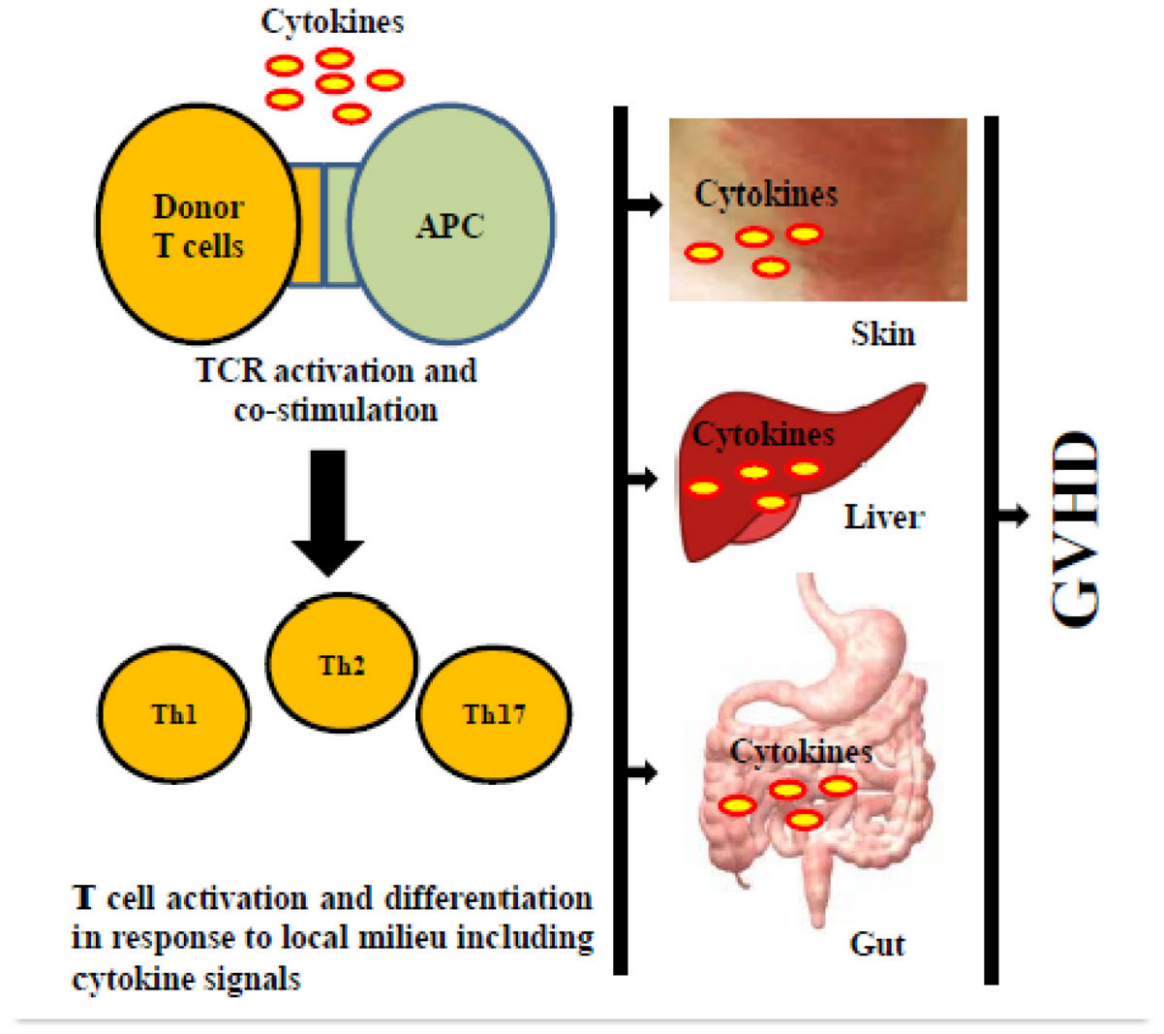
A graphical representation for the role of cytokines in GVHD pathogenesis. Following allogeneic hematopoietic cell transplantation, donor T cells recognize allo-antigens present on the host antigen presenting cells (APCs). As a result of T cell receptor (TCR) activation and co-stimulation, donors T cells activate, proliferate and expand in the presence of cytokines. The presence of various cytokines also influences the differentiation of naïve T cells into Th1, Th2, Treg and Th17 cells. Furthermore, T cells migrate to the target organs such as skin, liver and gut and release proinflammatory cytokines that lead to induction of GVHD.

Graft-versus-host disease (GVHD) is serious complication after allogeneic hematopoietic cell transplantation (Allo-HCT). During anallo-HCT procedure, hematopoietic cells harvested from an allogeneic donor are transplanted into a patient who suffers a certain type of hematologic malignancy or other hematologic or immunologic disease. After transplantation, however, donor derived immune cells may recognize normal host cells/tissues as foreign and subsequently cause tissue damages leading to onset of GVHD. The common symptoms of acute GVHD include weight loss, alopecia (hair loss on scalp and elsewhere on body), skin lesions, gastro-intestinal (GI) tract and liver complications that may lead to death [1]. Among various cellular immune components, T cells are crucially important in the pathogenesis of GVHD. Donor T cells undergo activation, proliferation and migration into target organs as a cascade of molecular events involving interaction between T cell receptors (TCR) and major histocompatibility complex (MHC) bound allo-antigens, co-stimulatory signaling and cytokine signaling. Cytokines are immune effectors as well as regulators of various immunologic conditions including inflammation and hypersensitivity reactions [2,3]. Many studies have demonstrated that cytokines are involved in various inflammatory and regulatory activities during GVHD [4,5]. Following encounters between donor T cells and host antigen presenting cells (APCs) presenting allo-antigens, allo-reactive donor T cells are activated and undergo vigorous proliferation and then migrate to the target organs and tissues. These events are accompanied with enhanced secretion of proinflammatory cytokines that contribute to severe acute GVHD. That is why a rapid onset of acute GVHD is considered as a result of “cytokine storm” [6]. The manifestation and severity of GVHD ascribe to naïve T cells maturing and differentiating into different lineages and phenotypes including regulatory T cells (Treg), Th1, Th2, and Th17 cells (Figure 1). This differentiation is associated with the presence of local cytokines that activate transcription factors and determine supremacy of certain phenotypes [7]. Th1 cells secret several cytokines namely interleukin-2 (IL-2), IL-10, interferon-gamma (IFN-γ), tumor necrosis factor-alpha (TNF-α), and TNF-β. Th2 cells secret cytokines like IL-3, IL-4, IL-5, IL-10, IL-13, IL-17E and IL-31. Th17 is known to secrets IL-17 mainly [7]. In GVHD, donor naïve CD4+ T cells recognize allo-antigens presented on the host’s APCs and differentiated into Th1, Th2, and Th17 cells depending upon local cytokine milieus.

In MHC-mismatched models of GVHD, T helper (Th) cells differentiated into Th1 subtypes that mediate tissue damage in GI tract and liver. In the absence of IFN-γ, Th cells differentiated into Th2 and Th17 subsets and caused damage in lung and skin. If there is neither IL-4 nor IFN-γ the dominating subset is Th17 cells that lead to tissue damage in skin. If both the IFN-γ and IL-17 are absent, the dominating subset is Th2 cells that cause idiopathic pneumonia in GVHD patients [8]. Furthermore, in the presence of IL-12 Th cells differentiate into Th1 phenotype and cause release of IFN-γ and IL-2 cytokines [9]. In summary, the literature has described cytokines as effector molecules as well as regulatory factors in GVHD pathogenesis. This review aims to corroborate the past and current knowledge regarding the functions of cytokines in GVHD pathogenesis with special emphasis on potential therapeutic managements.

### Th1 Cytokines in GVHD

Among various Th1 cytokines, IL-2 is one of the most studied cytokines for its role in activation, proliferation and expansion of T cells in GVHD. Several studies indicate a potential role of IL-2 in GVHD [10–14]. The role of IL-2 in GVHD is very diverse that includes amplification of allogeneic immune response, activation of T cells and NK cells, stimulating secretion of TNF-α by macrophages and inflammatory damages to the skin and gut [15]. Considering the importance of IL-2, high dose and low dose of IL-2 therapy are used to diminish GVHD [11,12]. Administration of high dose IL-2 for several days beginning on the day of allo-HCT attenuates GVHD mortality in lethally irradiated mice [11]. Low dose therapy with IL-2 has also been reported, that expands Treg population in vivo and is associated with a lower incidence of GVHD [12]. Low dose of IL-2 restores CD4^+^Foxp3^+^Treg homeostasis without causing any adverse effect on the graft-versus-leukemia/lymphoma (GVL) response [13]. On the other hand, prevalence of chronic GVHD is characterized by constitutive phosphorylation of Stat5 in conventional CD4+ T cells (Tcons) associated with elevated amounts of IL-7 and IL-15 and relative functional deficiency of IL-2. The IL-2 therapy resulted in the selective increase of Stat5 phosphorylation in Treg and a decrease of phosphorylated Stat5 in Tcons [14]. However, there are certain limitations of IL-2 therapy in GVHD. For examples, in experimental GVHD model, IL-2 administration to donor mice induces a dose-dependent expansion of Treg cells in the graft but is insufficient to suppress GVHD [16]. In xenogeneic model of GVHD where human peripheral blood mononuclear cell transplanted into immunodeficient mice, although, low-dose IL-2 administration caused increase in Treg cells but was unable to control pro-inflammatory cytokines production by pathogenic Tcons [16]. Taken together, studies are divided into pro- and anti-IL2 therapy in GVHD.

Another member of Th1 secreted cytokines, IL-10 (also known as human cytokine synthesis inhibitory factor) is a regulatory cytokine that play an important role in GVHD. IL-10 can modulate CD4+ T cells functions by down-regulation of another Th1 cytokine IL-2 [17]. IL-10 does not contribute to GVHD mediated by effector T cells. In contrast, IL-10 creates tolerogenic environment to allo-antigens independent of IL-2 or CD28 stimulation [18]. A study, using IL-10 deficient donor or host mice in a MHC-mismatched model of acute GVHD, reported increased GVHD if either donor or host B cells were unable to produce IL-10 [19]. The induction of IL-10 release in host B cells attenuates acute GVHD in experimental model [20]. Furthermore, donor bone marrow graft and Treg-derived IL-10 are important for the donor Treg-mediated suppression of GVHD [21]. High frequency of donor cells producing IL-10 in response to host allo-antigen stimulation has been correlated with the absence of acute GVHD post allo-HCT. Conversely, low frequency of IL-10 response is strongly associated with more severe GVHD [22].

Another major Th1 cytokine, IL-12, produced mainly by dendritic cells and macrophages, is a heterodimeric cytokine that mediated cellular immunity [23]. The dimeric components of IL-12 are a heavy chain subunit p40 and a light chain subunit p35 that are critical for the functions of active IL-12 [23]. Interestingly, IL-12 shares a common p40 subunit with another cytokine IL-23. This subunit is important for driving Th1 differentiation and stabilization of Th17 phenotype. Result showed that both donor- and host-derived p40 is important for the induction of acute GVHD. The therapeutic efficacy of anti-p40 was evident by its ability to reduce acute GVHD [24].

Pro-inflammatory cytokine IFN-γ is mainly produced by activated T-cells, NKT cells and NK cells. IFN-γ inhibits GVHD in lethally irradiated mice receiving allo-HCT but promotes lethality in un-irradiated and sub-lethally irradiated recipients [25]. The extent of conditioning markedly affects the role of IFN-γ in GVHD lesions mediated by CD4+ T cells. For example, in a GVHD model using sub-lethal total body irradiation (TBI), the absence of IFN-γ is playing a protective role in GVHD, while in lethal TBI condition, loss of IFN-γ is associated with increased pathogenesis [26]. However, there is experimental evidence that IFN-γ may not be required in GVHD pathogenesis but can facilitate the GVL effects [27]. Further studies are required to explore IFN-γ involvement in GVHD for more in depth insight on its use as a therapeutic target.

Another cytokine from Th1 family that plays important role in the GVHD is TNF-α. It is directly involved in tissue damages by inducing apoptosis or necrosis of target cells and synergizes with cytotoxic T lymphocytes and NK cells [28]. Therefore, use of TNF-α antagonist has shown promising results in GVHD management. For example, Korngold et al., have investigated that the inhibition of TNF-α during HCT can diminish inflammatory GVHD reactions without hindering effective GVL response [28]. However, a recent study reported that TNF-α priming can enhance CD4^+^ Foxp3^+^ regulatory cell suppressive function to attenuates GVHD [29]. Also, another study indicated that GVHD control depends to production of TNF-α by T cells and expression of TNFR2 on regulatory T cells [30].

In summary, the roles of Th1 cytokines in GVHD pathogenesis are diverse yet not completely defined. Management of Th1 cytokines for GVHD prophylaxis and treatment appears promising, but optimization is still an issue that needs further effort.

### Th2 Cytokines in GVHD

Th2 cytokines can attenuate Th1 cytokines such as TNF-α and interrupt Th1 cytokine cascade post allo-HCT and therefore was once thought to lead to the suppression of GVHD pathogenesis [31]. Recently, transplantation of myeloid derived suppressor cells (MDSCs) has been reported to skew allogeneic T cell response toward Th2 cells and enhanced Th2-specific cytokines that caused suppressed GVHD [32]. However, other studies have suggested various cytokines of Th2 family are associated with GVHD. For example, IL-3 is a growth promoting cytokine involved in the differentiation and apoptosis of various hematopoietic cell types [33]. Up-regulated level of IL-3 is reported in a significant subgroup of patients suffering from extensive chronic GVHD [34]. Similarly, high-dosage of IL-3 accelerates GVHD and impairs survival of the host [35]. Another Th2 cytokine, IL-4 is a pleiotropic cytokine produced by activated T cells [36]. The IL-4 receptor (IL-4R) also binds to IL-13, that contribute to several overlapping functions of IL-4 and IL-13 [37]. IL-4 plays an important role in the regulation or pathogenesis of allogeneic responses [38]. Th2 cell therapy can rapidly ameliorate severe GVHD by creating hindrance in IL-4 and IL-10 functions, IL-2 consumption and APC modulation [39]. IL-5 is another member of Th2 cytokine family originally defined as a T-cell-derived cytokine that triggers activated B cells for terminal differentiation into antibody-secreting plasma cells at least in mice [40]. Elevated level of serum IL-5 is reported in acute GVHD [41]. The cytokines IL-5, IFN-γ, and TNF-α have been used as biomarkers of acute GVHD [42]. IL-13 is mainly involved in the allergic inflammation and other ailments including GVHD [3]. Therefore, association between IL-13 levels and acute GVHD may be exploited as a strong predictor of this disease. In summary, it is now clear that acute GVHD is not a purely Th1-type cytokine-driven response, but Th2-type cytokine such as IL-13 has also been involved in the pathogenesis of acute GVHD [42]. Therefore, the roles of these Th2 cytokines need further mechanistic clarification in GVHD pathogenesis and management to achieve optimum exploitation.

### Th17 Cytokines in GVHD

Th17 cells are known to release cytokines including IL-17and IL-22 [43]. Th17 cells synergize with naive T cells to induce lethal GVHD. In vitro polarized Th17 cells can induce higher GVHD leading to the severe skin and pulmonary conditions [44]. Th17 cells have an inverse relationship with Treg cells In GVHD. The dynamic changes of Th17 and Treg cells along with the level of Th17 cytokines are associated with the onset and resolution of acute GVHD [43]. IL-17 mediates its function via surface receptors on target cells [45]. Participation of IL-17 has been studied in the acute rejection of organ transplants and GVHD [46]. Both CD4+ IL17-producing T cells and CD8+ IL17- producing T cells secret the IL-17 cytokine. These subsets of T cells are suspected to initiate Th1 response at early phase in GVHD [47]. In an allo-HCT model using pan T cells, IL-17 is dispensable for GVHD and GVL activity. However, IL-17 contributes to the early development of CD4+ T cell-mediated GVHD by up-regulating production of proinflammatory cytokines [46]. Concurrent to this study reports IL-17-producing CD8+ T (Tc17) in the initiation of GVHD [48]. In addition to IL-17, other Th17 cytokines are also targeted by researchers to explore their role in GVHD. Interleukin-22 protects intestinal stem cells from immune-mediated tissue damage and regulates sensitivity to GVHD [49]. Treatment with IL-22 in vivo after allo-HCT enhanced the recovery of intestinal stem cells, increased epithelial regeneration and reduced intestinal pathology and mortality from GVHD [50]. In addition, IL-22 producing RORγt+ILC3 subset was reported to be involved in the prevention of intestinal GVHD via strengthening the intestinal mucosal barrier [51]. Furthermore, one of the other important cytokines that is produced by Th17 and T follicular helper cells is IL-21 [7]. Also, splenic neutrophils can express IL-21 during acute GVHD [52]. IL-21 was shown to play a critical role in GVHD development through increasing B cell activation and proliferation, alloantibody generation and disrupting Treg homeostasis [53,54]. It was also shown that direct or indirect (through Rho associated kinase 2) inhibition of IL-21 ameloraited GVHD symptoms [55]. Taken together, these studies show that the diverse roles of Th17 cytokines in GVHD are highly important, yet more in depth explorations are still required to define the precise contributions of various Th17 cytokines in GVHD.

### Other Cytokines, Chemokines and T Cell Populations in GVHD

Apart from Th1, Th2 and Th17 cytokines, various other cytokines also play important roles in GVHD directly or indirectly. For example, TGF-β-dependent CD103 expression is involved in regulating destruction of gut epithelium by CD8+ T cells during GVHD pathogenesis [56]. The anti-inflammatory cytokine IL-35 can suppress acute GVHD in patients post allo-HCT. This cytokine targets phosphorylation of STAT1 and STAT4 which is generally inhibited in mice in acute GVHD. Treatment of IL-35 leads to up-regulated phosphorylation of STAT1 and STAT4 and amelioration of acute GVHD. These observations advocate the potential therapeutic efficacy of IL-35 in GVHD [57]. Homeostatic interleukin IL-7 regulates T cell survival and proliferation *in vivo* and is also known as a thymotropic cytokine along with SCF [58,59]. A recent study suggests that elevated IL-7 but not SCF is associated with development of GVHD [59]. Another proinflammatory cytokine, IL-15, induces T cell proliferation and demonstrates IL-2-like properties [60]. Both IL-7 and IL-15 have been associated with the peripheral T cells regeneration in mice and humans [61]. Considering the importance of IL-7 and IL-15 in immune functions, these cytokines have been targeted for the treatment of acute GVHD [61]. In addition, increased levels of cytokines and chemokines including B cell activating factor (BAFF), IL-33, CXCL10 and CXCL11 are reported in GVHD pathogenesis [62]. The role of chemokine CCR7 in GVHD is especially affiliated with gastrointestinal (GI) tract complications. CCR7 significantly regulates elevated allo-antigens presentation in mesenteric lymph nodes of GI tract [63]. Furthermore, the binding of IL-33 to receptor “suppression of tumorigenicity 2 (ST2)” presents intriguingly both pro-inflammatory and anti-inflammatory effects. The increased levels of soluble ST2 are a biomarker for steroid-refractory GVHD and mortality. Blockade of IL-33 and ST2 interaction induces marked reduction in GVHD lethality [64]. Another recent study suggests a role of IL-26 in the pathogenesis of transplant-related obliterative bronchiolitisas IL-26^+^CD26^+^CD4^+^ T cells in part induces chronic GVHD of the lungs [1]. Moreover, IL-1β and associated MyD88 signaling in dendritic cells and T cells are involved in GVHD. After conditioning therapy, the microbial products and uric acid can activate NLRP-3 in donor T cells to increase IL-1β expression that subsequently enhances GVHD severity [65]. On the other hand, it has been demonstrated that MyD88 deficiency in T-cell depleted donor BM leads to attenuated GVHD symptoms [66].

In addition to Th1, Th2 and Th17 cells that produce associated cytokines and affect GVHD, there are several important T cell populations that also impact GVHD via cytokines and other mechanisms. Many studies have documented the roles of Treg cells in GVHD. Both natural and induced Treg cells are able to restrain conventional T cell proliferation and attenuate GVHD with multiple mechanisms including IL-2, IL-10 and TGF-β [67]. CD4^+^CD103^+^Fop3^+^natural Treg can directly migrate to GVHD target organs decreasing disease severity [68]. Notably, the Th17/Treg ratio is correlated with clinical and pathological GVHD and therefore could be used as a biomarker of GVHD [69]. IL-2 treatment in combination with rapamycin has been shown to mitigate acute GVHD lethality, which is associated with increased expansion of donor-type CD4^+^Foxp3^+^ Treg cells and reduction of CD4^+^CD25^−^ conventional T cells [67]. In addition, T follicular helper (Tfh) cells are responsible for naïve B cells differentiation to memory B cells and immunoglobulin class switching. Tfh cells express BCL-6 as transcription factor and CXCR5 and PD-1 surface markers with high secretion of IL-21 cytokine. Tfh cells mostly locate in germinal center but a subset of Tfh cells have been detected in peripheral blood. Tfh cells are required for generation and maintenance of germinal center and B cells function in chronic GVHD. These circulating Tfh cells appear to cause aggravation of chronic GVHD [70,71]. Furthermore, a more recent study has described a new CD4^+^ memory T population with high expression of CD11c and α4β7 in gut that plays a pivotal role in initiating gastrointestinal GVHD via promoting Th1 response and cytokine production [72].

Taken together, these studies indicate that a wide range of cytokines and T cell populations are involved in the pathogenesis of GVHD. A thorough understanding of the complex molecular and cellular networks will provide better insights for more effective GVHD management.

### Updates on Cytokine-Related GVHD Management Strategies

Recent years witnessed a significant growth in findings related to the role of various cytokines in the pathogenesis and management of GVHD. Several new drugs have been used to treat GVHD based on cytokine regulation. For example, zinc supplementation was reported to be beneficial in the induction of tolerance that amelioratesTh1-dominated allogeneic immune response [73]. A recent study also advocates granzyme B (GzmB) based therapeutic approach because GzmB knockout T cells are associated with the production of prominent quantity of proinflammatory cytokines that exacerbated GVHD [74]. An *in vitro* study using dendritic cell (DC) culture indicated that bortezomib can inhibit the proliferation of DCs and also blocked expression of costimulatory molecules CD80 and CD86. This drug was also found to diminish IL-12 and TNF-α release in DCs following treatment of LPS [75]. Another study was carried out on valproic acid, a histone deacetylase inhibitor that also possesses anti-inflammatory effects. In MHC-mismatched transplantation mouse model, valproic acid down regulated Th1 and Th17 cell responses and cytokine production *in vitro* and *in vivo* [76]. The use cyclopentylamino carboxymethylthiazolylindole-7 (NecroX-7) is found useful in controlling GVHD in preclinical models. NecroX-7 is an inhibitor of chromatin protein high mobility group box 1 (HMGB1). NecroX-7 protects mice against lethal GVHD by reciprocal regulation of Treg/Th1 cells [77]. Erlotinib, an EGFR tyrosine kinase inhibitor ameliorates sclerodermatous GVHD. These beneficial effects were mediated by decrease in IFN-γ and IL-13 production and autoimmune B-cell activation [78]. Moreover, BET bromodomain inhibition can suppress GVHD through NF-κB regulation and decrease production of inflammatory cytokines such as IL-6, IL-12, TNF-α in DCs and IFN-γ, IL-2, IL-4 and IL-17 in activated T cells [79]. Although initial outcomes of these cytokine-related therapeutic regimens raise hopes for better treatment, further study is warranted to achieve optimum benefits.

Micro RNAs (miRs) play important roles in regulation of various immune responses such as infection, tumor, and autoimmunity [80]. Recently, the function of miR-17-92 cluster in allogeneic T cell response has been studied. Results suggest an important role for miR-17-92 in donor T cells for GVHD induction. The miR-17-92 promotes CD4+ T cell functions, Th1 differentiation, but down-regulates Th2 and inducible Treg functions [80]. In addition, miR-142 is implicated in hematopoietic functions including T cell response. *In vivo* deletion of miR-142 does not affect T cell development. But in vitro and multiple models of GVHD targeting miR-142 leads to attenuated T cell proliferation [81]. Recently, miR-155, known to regulate the innate immune system, is studied for its role in DC functions during GVHD. Study suggests that miR-155 deficiency in host system is associated with decreased pro-inflammatory cytokines and attenuated GVHD pathogenesis [82]. Moreover, an earlier study showed that expression of miR146a increased in donor T cells after transplantation and deficiency in Mir146a caused enhanced GVHD through TRAF6/TNF signaling pathway [83]. These studies suggest that genetic manipulation by targeting miRs in GVHD therapy may have beneficial impacts.

Uses agonistic antibodies have shown proved efficacy in GVHD management. Recently, αDR3, an antibody to death receptor 3 (DR3), has been investigated for its role in the management of GVHD. DR3 is mainly present on Treg cells, lymphoid tissue inducer cells and NKT cells [84]. Studies showed that agonistic antibody αDR3 expanded CD4+FoxP3+ Treg cells population *in vivo*. Apart from the expansion of Treg cells, αDR3 also down-regulated proinflammatory cytokines such as IFN-γ, IL-1β, and TNF-α. Notably this GVHD alleviating effect was achieved by a single dose of αDR3 [84]. Another target aimed to alleviate GVHD is the TNF-like weak inducer of apoptosis (TWEAK)/fibroblast growth factor-inducible 14 (Fn14) axis. It was observed that attenuation of TWEAK/Fn14 system alleviated disease development in several models of colitis [85]. In GVHD, proinflammatory cytokine TNF play very important role in intestinal cell death. TWEAK up-regulates TNF-induced cell death. Therefore, proper understanding of Fn14 in TNF associated GVDH pathogenesis will be useful. As such, an antibody-dependent cell-mediated cytotoxicity (ADCC)-defective Fn14-blocking antibody has been utilized by researchers to attenuate GVHD successfully [85]. These studies advocate promising roles of monoclonal antibodies in GVHD management.

Cytokines also find uses as biomarkers for GVHD. For example, tear cytokine profile was found associated with systemic chronic GVHD. This finding is evident by the presence of enhanced levels of IL-2, IL-10, IL-17α, IFN-γ, IL-6, and TNF-α tear cytokines in chronic GVHD patients [86]. Recently, IL-6 and IL-9 have been studied for their potential as biomarkers in GVHD [87]. In addition to modulating T cells, recent study has also shown a potential role of cytokines in modulation of NK cells. Adoptive transfer of IL-12, IL-15 and IL-18 pre-activated NK cells showed suppression of GVHD in a mouse model of MHC-mismatched HCT [88]. Together, adoptive transfer of cytokines pretreated immune cells will pose a potential impact on the GVHD management.

## CONCLUDING REMARK

In summary, the role of cytokines in GVHD is indispensable. Several therapeutic interventions based on targeting cytokines have already shown promising results in GVHD amelioration. As Th1, Th2 and Th17 cytokines are associated with the pathogenesis and management, exploitation of these immune mediators will produce practical approaches to combat GVHD. Further studies are required to understand the underlying mechanisms associated with the roles of cytokines in GVHD in order to achieve optimum diagnostic and therapeutic benefits.

## Abbreviations

Allo-HCT: Allogeneic Hematopoietic Cell Transplantation
Allo-HSCT: Allogeneic Hematopoietic Stem Cell Transplantation
BMT: Bone Marrow Transplantation
IL: Interleukin
TNF-α: Tumor Necrosis Factor Alpha
IFN-γ: Interferon Gamma
TGF: Transforming Growth Factor
GVHD: Graft-Versus-Host Disease
